# 3D printed polymers that mimic the mechanical properties of atherosclerotic blood vessels for training models: the advantageous degradation induced by UV radiation and hydrolysis

**DOI:** 10.1186/s41205-025-00288-5

**Published:** 2025-07-02

**Authors:** Joana Filipa Henriques, Lino Gonçalves, Ana Martins Amaro, Ana Paula Piedade

**Affiliations:** 1 Universidade de Coimbra, CEMMPRE – Departamento de Engenharia Mecânica, Coimbra, Portugal; 2 Universidade de Coimbra, Faculdade de Medicina, Serviço de Cardiologia do CHUC, Coimbra, Portugal

**Keywords:** Atherosclerotic Vessel, Additive Manufacturing, Dynamic Tensile Test, Stereolithography, Patient-Specific Models, UV degradation

## Abstract

**Background:**

Atherosclerosis is a chronic disease characterized by the narrowing and hardening of arteries that may induce serious complications and even sudden death. Percutaneous angioplasty is performed as the main treatment of atherosclerotic-based cardiovascular diseases, which are the leading cause of mortality worldwide. Patient-specific physical models of these vascular conditions would greatly assist percutaneous angioplasty medical training and planning. Such models must be composed of materials that accurately replicate the properties of tissues. However, this mimicking can be challenging due to the complexity and composition of atherosclerotic vasculature. As additive manufacturing allows the production of complex and personalized structures, it provides great potential for manufacturing those models. The application of additive manufacturing in this context is often associated with high production costs, mainly related to material synthesis. Commercial materials could break this limitation, but they are still misaddressed.

**Methods:**

Therefore, this work intends to explore the use of three different commercial UV-curable resins to mimic the several types of atherosclerotic vessels. They were manufactured by vat photopolymerisation process, specifically the stereolithography (SLA) technology to mimic atherosclerotic vessels. The mechanical performance of materials and the influence of immersion in phosphate buffered saline (PBS) solution and irradiation with UV light, during different times, were evaluated. Dynamic tensile tests were conducted to study the fatigue resistance of materials under physiological loads.

**Results:**

The results showed that immersion in PBS solution enhanced the dynamic mechano-stability. Likewise, irradiation with UV-C light was pointed out as an interesting strategy to adjust the hardness of materials, with the advantage of being a fast and low-cost approach.

**Conclusion:**

Comparisons with the literature supported that all used materials are suitable for mimicking the mechanical properties of atherosclerotic vessels, specifically when previously immersed in physiological-simulated fluids, such as PBS.

## Introduction

Anatomical models are structures that replicate human anatomy and pathology [1]. For centuries, they have played a crucial role in medicine due to their ability to demonstrate clinical features of diseases prior to surgical intervention or medical treatment [2]. Nowadays, with the development of biomedical imaging techniques, particularly in imaging acquisition and processing, the characteristics of patients’ organs have been generated with much more detailed information, improving diagnosis and providing higher insights into pathologies [3]. Although the resulting virtual models can provide three-dimensional (3D) anatomy and inter-tissue relationships, they do not offer tactile properties or the opportunity for physicians to train the surgical procedures [1]. Patient-specific physical models break down these limitations.

In this context, additive manufacturing, commonly known as 3D printing, is extremely advantageous due to the prospect of rapidly fabricating various and complex structures on a custom-made basis with minimal waste production [4]. Coupled with the progress of design, modelling, and scanning, 3D printing has gradually become an emerging and crucial adjunctive tool in medicine. Patient-specific models are used to assist in surgical planning and training, upgrade operative skills, and enhance doctor-patient communication, ultimately reducing the risk and duration of the surgical procedure. This strategy has been applied to neurosurgery [5, 6], thoracic [7, 8] and tracheal [9] interventions, urology and renal treatment [10–12], liver [13, 14] and other pathological abdominal conditions [15] as well as craniomaxillofacial procedures [16, 17]. Another field often addressed by medical 3D printing is the cardiovascular field. It is used to help the diagnosis and treatment of diseases like congenital heart disease [18], aortic aneurysm [19], cardiac tumour [20], and cardiovascular diseases (CVD), which are the leading cause of mortality worldwide [21].

The primary mediators of CVD burden and death trends are attributed to atherosclerotic-mediated CVD, such as ischemic heart disease and stroke [22]. Atherosclerosis is a chronic vascular disease characterized by the accumulation of cholesterol on the vessel wall, leading to the formation of rigid plaques that cause vessel hardening and problems with blood flow and oxygen supply [23]. Once formed, the plaque is covered by a fibrous cap, which rupture can lead to severe events, such as cerebrovascular accidents and myocardial infarction, and thus, sudden death [24]. Balloon angioplasty coupled with stent implantation is commonly performed to prevent the rupture of the fibrous cap and occlusion of the vessel lumen, thereby increasing the life expectancy of patients.

Procedural planning and training are of the most importance to improve the success of the intervention and reduce its time and inherent risk [25]. Additively manufactured physical models could assist these demands. For instance, Valverde and colleagues [26] 3D printed aortic models using two distinct polymeric materials (a polyurethane-based composite and poly(lactic acid) to determine the optimal stent length and avoid stent migration and other complications associated with aortic arch hypoplasia treatment. Although models allowed for the accurate replication of the patients’ anatomy, the biomechanical properties were not addressed [26].

To investigate the physiologically relevant forces, Stepniak and coworkers [27] used three different 3D printing technologies with materials with different hardnesses, namely: polyurethane (hardness 92 Shore A), Tango resins (hardness 75 and 61 Shore A) and “flexible” resin (hardness 73 Shore A) to obtain a computed-tomographic model of a coronary artery network with high geometrical accuracy for biomedical imaging. The anthropomorphic plaque mimics were valuable in designing optimal imaging protocols; however, the mechanical properties of the materials differed from those of biological tissues [27]. Multilayered 3D vascular replicas were also developed using indirect 3D printing [28]. 3D printed poly(acrylonitrile–butadiene–styrene) (ABS) sacrificial structures were dip coated with different hydrogels, resulting in an effective multilayered replica training platform. Nevertheless, some problems must be considered and addressed, namely the large amount of material waste, time consumption, and the intensity of labour [28].

Similarly, Gao group [29] has developed a cerebral artery surgery simulator. Despite the accuracy of 3D printed vascular structures in terms of anatomy and hemodynamic performance [29], their main purpose was tissue engineering and 3D cell culture, which are outside the scope of this work. None of the described works have considered the rigid calcified inclusions characteristic of atherosclerotic plaques advanced stages. In this frame, Izzo and coworkers 3D printed a heart model to plan the transcatheter mitral valve replacement [30]. The vascular anatomy was produced using Tango resin with a hardness of 26–28 Shore A and the calcification mimicked with another Tango resin with a hardness of 68–72 Shore A. The mitral valve presented calcifications that were 3D printed with a rigid material; however, the biomechanical and sensitive properties were not fulfilled, and radio-attenuation challenges must be overcome [30]. Therefore, and to the best of our knowledge, no work fully recapitulates the features and properties of atherosclerotic blood vessels.

Accordingly, we propose a preliminary study of materials to be used as atherosclerotic-mimicking materials. The process of Vat photopolymerization, particularly stereolithography (SLA) technology, nomenclature according to ISO/ASTM 52900:2021 standard for additive manufacturing, was used due to its high resolution and capability to print soft and hard materials. Moreover, since it directly applies additive manufacturing, it overcomes some challenges associated with material waste and time consumption. Three different flexible resins with distinct Shore A hardness were considered, to study their ability to mimic several stages of atherosclerotic plaque formation (presenting different hardness) and their static and dynamic mechanical properties were studied. The storage condition was also evaluated to explore the effects of immersion into an aqueous solution on the material properties. Consequently, this work could give important initial insights into the use of different materials to mimic the various components and stages of atherosclerotic disease.

## Materials and methods

### Materials

Three resins that produce polymers with Shore hardness values between 55 and 82 A were chosen due to their mechanical behaviour similar to an elastomer and were used without further modification. Liqcreate Flexible-X resin (Flex-X) with a Shore hardness of 55 A was obtained from Liqcreate® (Utrecht, The Netherlands). In turn, Flex 63 A resin and Flex 82 A resin, with Shore hardnesses of 63 A and 82 A, respectively, were provided by FormFutura® (Nijmegen, The Netherlands).

### 3D printing and post-processing

The resins were photopolymerized and 3D printed using an Anycubic® Photon Mono LCD SLA printer (Anycubic®, Shenzhen, China). The 3D printer has a 6.08’’ monochrome 2 k LCD screen with an in-plane resolution of 51 µm 405 nm wavelength radiation from a high-quality filament light. The printing parameters were previously optimised for each material (Table 1), and *g-code* files were created using the Anycubic® Photon Workshop v3.1.0 slicer software, developed by the manufacturer. The dimensions of 3D printed specimens varied depending on the specifications of the characterisation test, as described in the following sections.

**Table 1:** Printing parameters used for each resin

	**Flex-X**	**Flex 63A**	**Flex 82A**
Layer thickness	50 µm	50 µm	50 µm
Exposure time	12 s	4 s	6 s
Off time	1 s	4 s	1 s
Lift speed	20 mm·s^−1^	4 mm·s^−1^	1.5 mm·s^−1^
Bottom exposure time	80 s	45 s	40 s
No. bottom layers	2	3	3

After being 3D printed, the specimens were post-processed in the Anycubic® Wash & Cure Machine 2.0 (Anycubic®, Shenzhen, China) to remove any unreacted resin left on the surface and to cure the resin completely. The solid specimens were washed by immersing them in 96% (v/v) ethyl alcohol for 5 min, followed by a 10 min resting period and a curing step of 30 min under 405 nm irradiation.

#### Swelling and biodegradability

To assess the swelling capacity of SLA processed parts, four specimens of each material (10 × 10 × 2 mm^3^) were immersed in 10 mL of aqueous phosphate buffered saline (PBS) solution at pH = 7.4. Before the immersion, initial dry masses (${m}_{0}$) were registered (ADJ 100–4 Digital analytical scale from Kern® & Sohn GmbH, Sande, Germany), and the initial dimensions were measured with a calliper. Specimens were left under orbital shaking (100 rpm) at 37 °C (Incu-Shaker™ 10L from Benchmark Scientific®, New Jersey, USA). Swelling profiles were monitored by registering the mass (${m}_{t}$) and dimensions of specimens every 4 days for a total period of 70 days. The swelling capacity ($SC$) was determined according to Eq. 1.

1 $$SC (\%)=\left(\frac{{m}_{t}-{m}_{0}}{{m}_{0}}\right)\times 100$$

In turn, the degradation of 3D printed parts was assessed by measuring the mass lost during the immersion period and their dimensional change. Following the procedure of McBath and Shipp [31], the mass loss was determined by drying the specimens for several days and registering the final mass (${m}_{f}$).

### Chemical characterization

Fourier transform infrared spectroscopy (FTIR) was employed to analyse the primary chemical composition of the resins. Parallelepipedal specimens of 60 × 20 × 2 mm^3^ were 3D printed and post-processed. For each material, three specimens were characterized. The infrared spectra were acquired at 20 °C using a Bruker® Alpha III spectrometer equipped with an attenuated total reflectance (ATR) module featuring a platinum/diamond crystal, an RT-DLATGS detector, and a KBr beam splitter. Data acquisition was made using a 4 cm^‒1^ resolution with 24 accumulation interferograms. After data registration, the spectra were analysed using the OPUS® (version 8.5) vibrational spectroscopy software supplied by the manufacturer.

### Mechanical characterization

#### Static tensile test

**Fig. 1:**
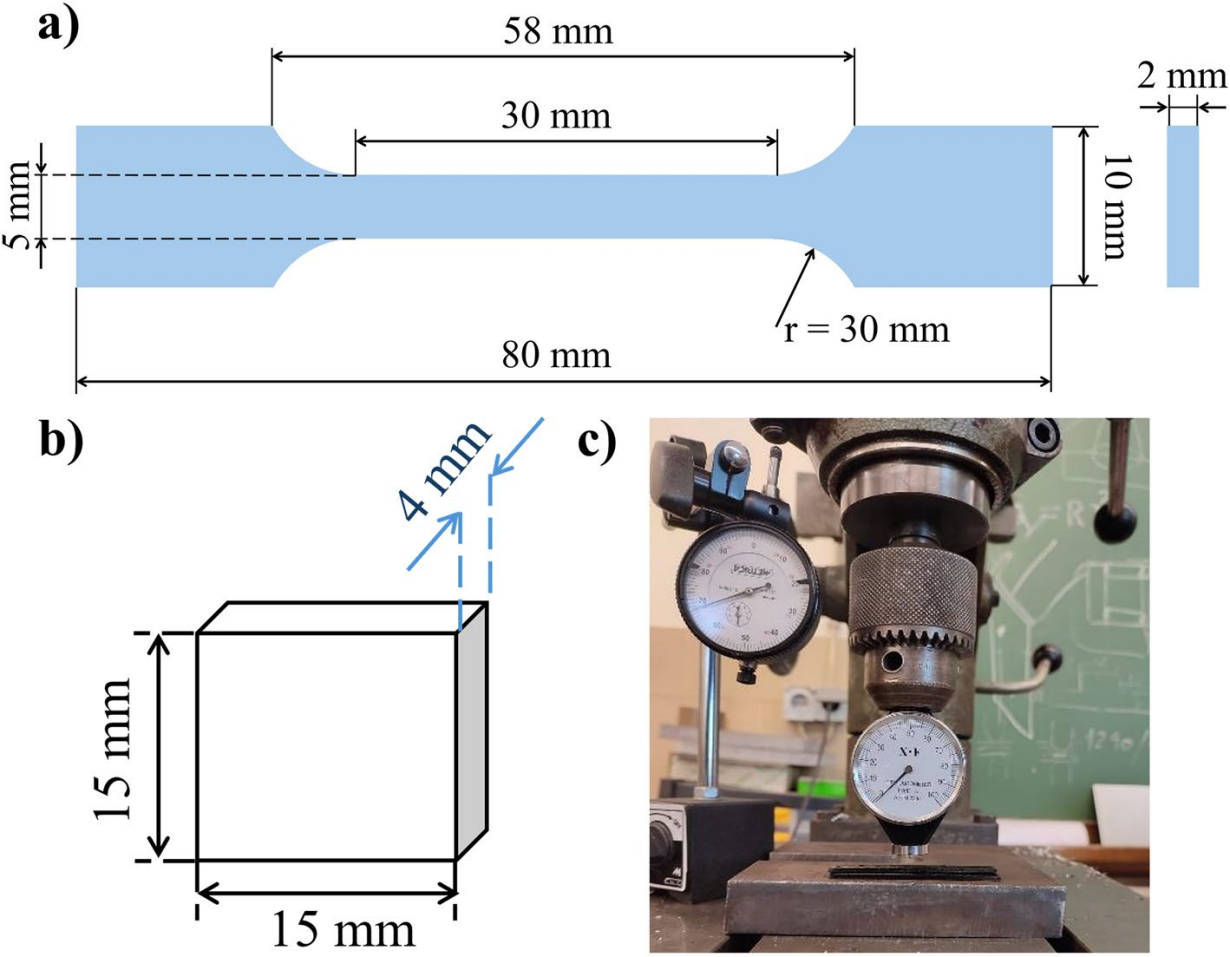
**a**) Dimensions of dog-bone shaped specimens used for tensile tests. The dimensions of these specimens (Type 1BA) are in accordance with the ISO 527 standard. **b**) and **c**) Measurement of Shore A hardness. **b**) Dimensions of 3D printed specimens used on the test; **c**) Equipment used to measure Shore A hardness

The mechanical properties of the cured polymers were determined through tensile tests using dog-bone shaped specimens with the dimensions described in ISO 527 standard [32] (Fig. 1 a)). Tests were conducted on a Universal Testing Machine (Instron® 6800 – Model 68TM-5, Massachusetts, USA) equipped with a 5 kN load cell and 1 kN pneumatic clamps (2.5 bar) at a 1 mm·min^−1^test speed. The gauge length was set to be 40 mm. Five specimens of each material were tested at room temperature (~ 20 °C). The results were analysed on Instron® Bluehill software provided by the manufacturer, and from the resulting stress–strain curves, it was possible to calculate the strain (${\varepsilon }_{b}$) and stress at break (${\sigma }_{b}$), tensile strength (${\sigma }_{m}$), and Young Modulus ($E$).

An additional five specimens of each material were immersed in PBS (pH = 7.4) and tested after 15 days to investigate the influence of storage conditions on their mechanical behaviour. Specimens were immersed for this period under orbital shaking (100 rpm) at 37 °C (Incu-Shaker™ 10L from Benchmark Scientific®, New Jersey, USA). Tests were performed at the same test conditions for the dried specimens.

#### Dynamic tensile test

Since this study aims to identify 3D printable materials that accurately mimic the mechanical properties of atherosclerotic blood vessels, enabling the production of biomodels, assessing their behaviour under pulsatile stresses is necessary. Therefore, the mechanical performance of materials under cyclic uniaxial tensile loads was also studied.

A cyclic tensile test was performed on six specimens of each cured polymer (three previously immersed in PBS solution) with the exact dimensions as those used in the static tensile test (Fig. 1 a)). The specimens were pre-loaded to 0.5 N at a strain rate of 1 mm·min^−1^ and then cyclically loaded and unloaded 100 consecutive times up to 0.67 N and down to 0.33 N, at a strain rate of 1 mm·min^−1^ strain rate. The edging force values were determined by calculating the circumferential stress sensed by artery walls (${\sigma }_{circ}$) under arterial pressures of 200 mmHg (worst case scenario for a high systolic pressure) and 100 mmHg (worst case scenario for a high diastolic pressure), according to the Eq. 2.

2 $${\sigma }_{circ}=pR/D$$

where $p$ is the arterial pressure, $R$ is the vessel radius, and $D$ is the thickness of the artery wall [33]. For a radius of 2.5 mm and a wall thickness of 1 mm, the equivalent circumferential stresses are 33 kPa and 67 kPa for arterial pressures of 100 mmHg and 200 mmHg, respectively. Furthermore, to determine the required force ($F$) to produce the same stress ($\sigma$) on dog-bone shaped specimens, a stress formula (Eq. 3) was applied.

3 $$\sigma =F/{A}_{0}$$

where ${A}_{0}$ is the initial cross-sectional area of specimens (5 × 2 mm^2^). The resulting forces were 0.33 N and 0.67 N for 33 kPa and 67 kPa, respectively. Although it does not fully represent the pathophysiological condition, it is a good starting point. In fact, in physiological conditions, blood vessels are subjected to internal and external forces. The former is caused by blood (shear stress) and pressure (circumferential and axial stresses), applicable for tubular configurations, and, thus, influenced by geometrical parameters of the vessel (wall thickness, radius, length) [33]. Moreover, blood vessels are multi-layered structures with the mechanical behaviour of the vessel wall being dominated by elastin fibres or rigid collagen fibres for lower and higher blood pressures, respectively [23]. Although the test conditions do not fully represent the pathophysiological environment, they are a good starting point for testing the dynamic properties of the studied materials.

Tests were conducted at room temperature (~ 20 °C) using the Instron® 6800 universal testing machine and acquired through the Instron® Bluehill software.

#### Shore a hardness measurement

Shore A hardness of materials was determined. Triplicates of each resin were manufactured with 15 × 15 × 4 mm^3^ (Fig. 1 b)), using the printing parameters previously described. Hardness measurements were conducted using an X.F. Type A hardness tester durometer (ViaIndustrial®, Bogotá, Colombia), according to the ASTM D2240 standard [34]. The measurements were carried out on an operating stand (Fig. 1 c)). The holding time was as low as possible, approximately 2 s, to minimize creep effects that could underestimate the registered values [35]. Each specimen/replica was tested five times. The results are presented as mean ± standard deviation.

A set of specimens (three per cured polymer) was immersed in PBS solution and left under orbital shaking at 37 °C for 15 days to evaluate the effect of storage conditions. The measurements were conducted following the same procedure.

In order to determine if the Shore A hardness values can be tuned by changing the processing procedure, specimens of 20 × 20 × 4 mm^3^ were exposed to ultraviolet-C (UV-C) light (6 W lamp, Germix® UV-C sterilizer, China) for 30 and 60 min, and the Shore A hardness values were measured. Two specimens were used for each testing time, and five measurements were made per specimen. The values are presented as mean ± standard deviation values.

### Surface characterization

The static contact angle of the processed materials was determined using the optical tensiometer Attention® Theta Flex (Biolin Scientific®, Västra Frölunda, Sweden) via the sessile drop method. Five drops of distilled water (4 µL) were deposited at 20 °C on each specimen (60 × 30 × 2 mm^3^ – one per polymer). The contact angle was automatically determined by the OneAttention® software (version 4.0.6) provided by the manufacturer, following the Laplace-Young model [36, 37].

Wettability and surface tension are significantly affected by surface roughness. Consequently, the surface topography was assessed using the Attention® 3D Topography Module, which is included in the optical tensiometer. The topography is analysed from images taken by an incorporated camera by projecting light patterns onto the surface, and its shape is reconstructed. The structured lighting phenomenon (fringe projection phase-shifting method) enables the measurement of 2D and 3D roughness parameters that give detailed surface topographical characterization. Thus, the main roughness parameters ${S}_{dr}$ (ratio between the interfacial and projected area), *S*_*a*_ (arithmetic average height of the surface), ${S}_{q}$ (root mean square height of surface area), and the true to projected surface area ratio (*r* factor, $r=1+\frac{{S}_{dr}}{100}$) were measured and used to calculate the corrected contact angle. According to Wenzel’s equation (Eq. 4) the apparent contact angle (${\theta }_{a}$ – measured value) is given by:

4 $$cos{\theta }_{a}=r\times cos{\theta }_{r}$$

where *r* is the true projected area ratio (*r* factor determined in topographic analysis) and ${\theta }_{r}$ is the contact angle for a roughness-free surface, also known as Young’s contact angle [38].

### Morphological characterization

The surface and cross-sectional morphologies of the processed materials were assessed using Scanning Electron Microscopy (SEM) with a Hitachi® SU3800 microscope (Hitachi®, Tokyo, Japan) operating at an accelerating beam voltage of 5 kV and an emission current of 57 µA. Before the microscopy analysis, specimens were coated with a thin gold layer (~ 30 nm) via the sputtering technique for 250 s, using the 108 Auto Sputter Coater (Cressington® Scientific Instruments, Watford, UK). This coating was applied to enhance the electrical conductivity of polymeric specimens.

The cross-section analysis was performed after tensile rupture during tensile tests. The surface of the XZ plane of untested specimens was also observed.

## Results and discussion

### Chemical analysis

**Fig. 2:**
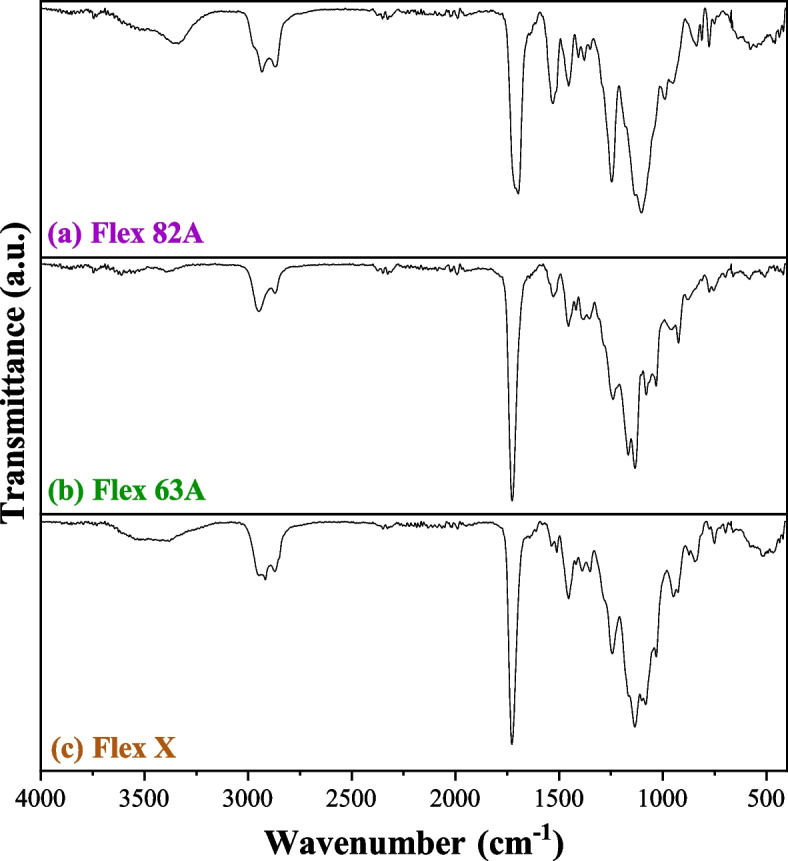
FTIR spectra of post-processed specimens (**a**) Flex 82 A resin, (**b**) Flex 63 A and (**c**) Flex X

The FTIR spectra of the post-processed polymers are presented in Fig. 2. All spectra show a double peak at 2947 cm^−1^ and 2871 cm^−1^ corresponding to C-H stretching vibration [39], followed by a highly intense peak between 1730 cm^−1^ and 1700 cm^−1^, derived from the stretching of C = O bonding from the ester functional groups [40–42]. The combination of these two vibration bands is related to the presence of both alkyl ester and methacrylic ester [41] in the structure of the studied resins, which is according to the chemical composition of the materials provided by the suppliers [43–45].

Additionally, the double band at 2850 and 2990 cm^−1^, in association with a weak transmission intensity at 1380 cm^−1^, followed by another band of greater intensity at 1460 cm^−1^, is attributed to the presence of methyl groups, confirming that all the analysed materials contain C-H bonds [46].

It is also notable that the FTIR spectra of Flex X and Flex 63 A have identical profiles within the wavenumber interval of 2000–750 cm^−1^. This similarity aligns with their formulation, which is mainly constituted by urethane oligomers polymerised and cross-linked through SLA technology. The intense band at 1242 cm^−1^ is associated with the stretching vibration C–O–C in -NHCOO- [47], while the region between 1270 and 950 cm^−1^, where it is observed a profile with several bands, is due to in-plane bend vibration of C-H derived from aromatic functional groups, appearing in urethane polymers [48]. Likewise, the double peak at 1080 and 1030 cm^−1^ is due to the stretching vibration of primary amine [48].

In turn, according to the supplier information [45], Flex 82 A is obtained from methacrylic and ether oligomers. Therefore, the high intense peak at 1530 cm^−1^ from the stretching vibration of the ether group [49] is explained by the presence of 2,2’-ethylenedioxydiethyl dimethacrylate.

Another point worth considering is the absence of signals between 1650 and 1600 cm^−1^, attributed to C = C bonds in acrylate monomers [41, 42, 50, 51]. This absence suggests that the double bonds were utilised in the formation of the cross-linked network during photopolymerization [50].

### Swelling capacity and biodegradability

The area of biomedicine, particularly the field of biomedical devices, must always consider the effect of physiological solutions on the final purpose of a material. The contact between the devices and biological fluids is constant, so it is imperative to predict how the material will behave once immersed in physiological fluids, which are mainly composed of water. Specifically, since the envisaged application is related to the production of cardiovascular models to simulate interventional procedures, studying conformational and mass changes due to immersion in simulated body aqueous solutions would be critical to determining the useful lifetime of the models after cyclic passages of simulated body fluids.

**Fig.3:**
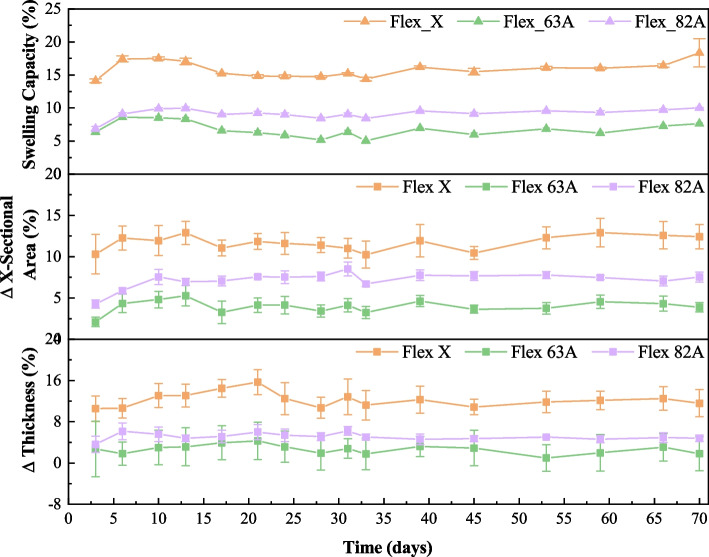
Effects of immersion in PBS solution (37 °C, 100 rpm) on the swelling capability, cross-sectional area, and thickness of post-processed specimens (Flex X, Flex 63 A and Flex 82 A)

Therefore, to assess the swelling capacity of the selected SLA-processed materials, four specimens (10 × 10 × 2 mm^3^) of each resin were photopolymerized and immersed in PBS solution, with their masses being systematically measured. PBS solution mimics the physiological conditions of the human body, so swelling and degradation were studied under these conditions [31]. After $t$ days of immersion, the swelling capacity was calculated, and the resulting swelling profiles were recorded over 70 days, as shown in Fig. 3.

The swelling capacity of all three materials increased sharply through the first five days. During the next ten days, their capacity remained relatively unchanged, and a decreasing tendency was observed after the initial 15 days, with a plateau on the final days. The decrease in swelling occurring on the 15th day could be due to the degradation of the polymeric chains, which jeopardises the water retained in the bulk. From now on, it will be considered that the maximum swelling capability will be reached after 15 days of immersion.

By comparing the studied resins, it can be established that Flex X (17.49 ± 0.23%) and Flex 82 A (9.97 ± 0.19%) have the highest swelling capacity. This observation can be explained by the higher content of -OH groups [52, 53], confirmed by FTIR spectra (Fig. 2 a) and Fig. 2 c)), in which there are two broad bands at 720–590 cm^−1^ and 3400–3200 cm^−1^, associated with out-of-plane bend and stretching vibration of -OH group [48], respectively. On the other hand, Flex 63 A specimens have swollen less (6.56 ± 0.22%). This could be related to the cross-linking percentage [53–55]. While the other resins have functional monomers ((5-ethyl-1,3-dioxan-5-yl) methylacrylate and (2,2’-ethylenedioxydiethyl) dimethacrylate, in Flex X and Flex 82 A, respectively) that enable higher cross-linking percentages, Flex 63 A has not, which diminishes the cross-linking percentage and, thus, less water retention.

The dimensions of the specimens were also monitored, namely the thickness and the cross-sectional area (Fig. 3 and Table 2). Despite the wide dispersion of values, which is reflected in the large standard deviations, the variations in thickness and cross-sectional area after 70 days of immersion (Fig. 3) are similar to the corresponding swelling capacities, as expected. The water entrapped in the porosities of the specimens induced measurable dimensional variations.

**Table 2:** Degradation and dimensional variations of the cross-linked polymers after 70 days of immersion into PBS solution, followed by 5 days of dry (50 °C)

Material	Degradation (%)	Thickness Variation (%)	Cross-Sectional Area Variation (%)
Flex X	1.34 ± 0.10	↑ 2.12 ± 1.84	↑ 0.49 ± 0.92
Flex 63A	3.17 ± 0.12	↓ 1.38 ± 3.70	↓ 2.68 ± 0.66
Flex 82A	0.63 ± 0.11	↑ 1.17 ± 0.70	↑ 0.03 ± 0.66

Regarding degradability, the weight loss of Flex 63 A specimens impacted their dimensions, as evidenced by a decrease in both thickness and cross-section. In fact, despite the lower swelling capacity, the degradation was the highest (Table 2), which may be inconsistent. However, these results could be related to the leaching of unreacted monomers, dimers, and small oligomers from the surface into the aqueous environment due to erosion at the polymer-fluid interface [56].

Therefore, the results indicate that Flex 63 A specimens are most susceptible to sustaining mass losses and dimensional variations after immersion in PBS and subsequent drying, which can be attributed to the loss of intrinsic properties. On the other hand, Flex X and Flex 82 A are more sensitive to aqueous environments due to the increase in weight and dimensions upon immersion in PBS. These effects will be reflected in their mechanical performance once they are used in physiological conditions. Nevertheless, it is worth noting that compared with other materials with increased swelling capacities, such as cryogels and hydrogels [57, 58], the studied resins do not have significantly high swelling profiles, even after 70 days of immersion in PBS solution.

### Mechanical characterization

#### Static tensile test

Uniaxial tensile tests were conducted to determine the mechanical behaviour of the post-processed polymers. Since the envisaged application of the materials concerns in vivo-like environments, wet and dry specimens were tested. The wet specimens were immersed in PBS solution for 15 days (100 rpm, 37 °C) to achieve maximum swelling capabilities. PBS is a commonly used benchtop fluid to simulate the inorganic phase of human blood without inducing biohazard or shelf-life concerns associated with using true blood or blood components [59]. The tests were carried out until the specimens were fractured. The yield strength (${\sigma }_{y}$), ${\varepsilon }_{b}, {\sigma }_{m}$, and $E$ values were determined and summarized in Table 3. Due to the brittle behaviour of polymers, the first local maximum observed during the tensile test [32], i.e., the strength (${\sigma }_{m}$) corresponded to stress at break (${\sigma }_{b}$) for all specimens.

**Table 3:** Mechanical properties of the post-processed polymers from tensile tests. Results are presented as mean ± SD

**Material**	**Storage Conditions**	${{\varvec{\varepsilon}}}_{{\varvec{b}}}$**(%)**	${{\varvec{\sigma}}}_{{\varvec{m}}}\boldsymbol{ }({\varvec{M}}{\varvec{P}}{\varvec{a}})$	${{\varvec{\sigma}}}_{{\varvec{y}}}\boldsymbol{ }({\varvec{M}}{\varvec{P}}{\varvec{a}})$	${\varvec{E}}\boldsymbol{ }({\varvec{M}}{\varvec{P}}{\varvec{a}})$
Flex X	Dry	40.24 ± 10.75	0.91 ± 0.20	0.40 ± 0.16	2.72 ± 0.24
	Wet	11.34 ± 2.31	0.29 ± 0.05	0.25 ± 0.06	2.21 ± 0.56
Flex 63A	Dry	9.18 ± 3.94	0.33 ± 0.17	0.23 ± 0.11	3.35 ± 0.40
	Wet	9.13 ± 2.95	0.36 ± 0.11	0.23 ± 0.04	3.92 ± 0.56
Flex 82A	Dry	45.96 ± 3.54	5.27 ± 0.51	0.27 ± 0.06	34.60 ± 5.50
	Wet	6.16 ± 2.31	0.67 ± 0.30	0.42 ± 0.03	11.52 ± 2.21

From the results, it is concluded that the wetting process contributes to the decrease in the mechanical resistance of the materials. Young’s modulus and stress at break have significantly decreased for wetted specimens of Flex X (19% and 68% for $E$ and ${\sigma }_{b}$, respectively) and Flex 82 A (68% and 87%, for $E$ and ${\sigma }_{b}$, respectively). This reduction may be attributed to the plasticizer effect of water [60, 61]. In the Flex 63 A specimens, the wetting process has not resulted in any significant changes to their mechanical properties. As previously discussed, Flex 63 A specimens exhibit lower swelling capabilities, i.e., less water absorption, making the plasticiser effect of water almost unnoticeable.

It is also noteworthy that the Flex 82 A specimens have the higher Young’s modulus value, and Flex X specimens present the lower. This can be attributed to the functional monomers composing each resin: in the case of Flex X, urethane oligomers, associated with elastomeric behaviour, result in inferior $E$; the Flex 82 A, in turn, is made of methacrylic monomers that confer a more rigid structure, with stress–strain curves characteristic of thermosets, and then superior $E$ [50, 51, 62]. The mechanical behaviour of Flex 63 A specimens is somewhat different from the other two. As expected, the higher the Shore A hardness value provided by the supplier, the higher the $E$ value [63, 64].

Likewise, the addition of polyurethanes and soft rubber-like segments to resins contributes to the reduction of ${\sigma }_{m}$ [65], which also corroborates the differences between the polyurethane-based resins (${\sigma }_{m}$ of ~ 0.9 MPa and ~ 0.3 MPa for Flex X and Flex 63 A, respectively) and the polymethacrylic based resin (${\sigma }_{m}$ of ~ 5.3 MPa for Flex 82 A).

The mechanical properties of the materials studied in this work are consistence with those of atherosclerotic vascular plaques, as reported in the literature. For instance, Kobielarz and coworkers [66] have characterised human aortic atherosclerotic plaques using a uniaxial tensile test. They concluded that the stiffness of lipid, fibrotic, and calcified plaques were 3.50 MPa, 6.56 MPa, and 6.67 MPa, respectively, within the interval of elastic modulus obtained for Flex 63 A and Flex 82A. Flex X, Flex 63 A and Flex 82 A seem to be suitable to mimic atherosclerotic vascular tissues. By way of illustration, a work developed by Norris and colleagues [59] tested and compared four UV-cured polymers with human carotid arterial tissues. Tensile tests were conducted, and the results showed that polymeric materials had a higher tensile modulus (11 to 16 MPa) than the donated tissues (2 to 7 MPa), indicating some incompatibility between the materials and mimic these vascular tissues [59]. However, the materials used in our work yield similar results, primarily the Flex X and Flex 63 A polymers, which have a tensile modulus ranging from 2.5 to 3.5 MPa.

Moreover, the studied UV-cured polymers present values in the range of those of biological tissues regarding the ultimate tensile stress, i.e., ${\sigma }_{b}$. As published [67], diseased fibrotic media, fibrotic intima at the medial border, fibrous cap, and adventitia of human atherosclerotic plaques from iliac arteries exhibit ${\sigma }_{b}$ values ranging from 185 to 955 kPa, which is in the interval of values obtained for wet specimens of Flex X, Flex 63 A, and Flex 82A. The strength of human atherosclerotic plaques has been also assessed [68]. Predominantly fibrocalcific plaques and specimens predominantly constituted by lipid core atheroma showed ${\sigma }_{m}$ 0.22 ± 0.12 MPa and 0.49 ± 0.23 MPa, respectively [68]. The results confirm that the materials addressed in this work are suitable for mimicking these biological tissues. Nonetheless, reported stress failures of 0.51 MPa, 0.90 MPa, and 1.64 MPa for lipid calcified and fibrotic plaques in human aortas [66] are outside the range of those obtained for the studied materials. Even so, as the mechanical characterisation of biological tissues remains unstandardized, the variability of results still hampers direct comparisons between atherosclerotic tissues and biomimicking materials [23]. Therefore, cautious conclusions should be made.

### Dynamic tensile test

**Fig. 4:**
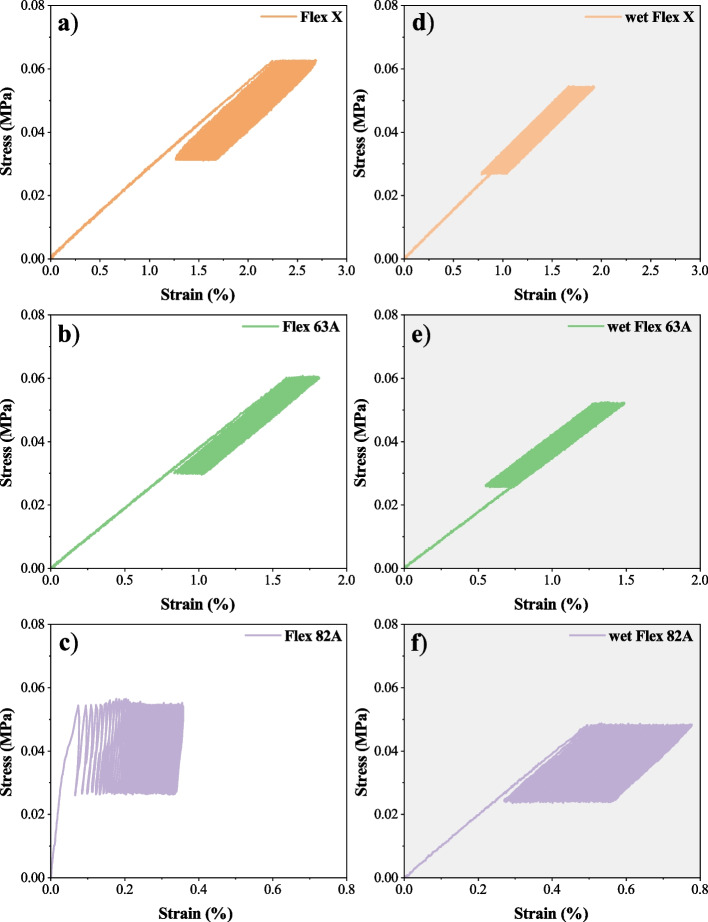
Representative stress–strain curves of cyclic tensile tests. **a**-**c** dry specimens; **d**-**f** specimens that were immersed into PBS solution for 15 days, to ensure the maximum swelling capability. The reader must pay careful attention to the different x-scales: **a**), **d**) 0–3; **b**), **e**) 0–2; **c**), **f**) 0–0.8

**Fig. 5:**
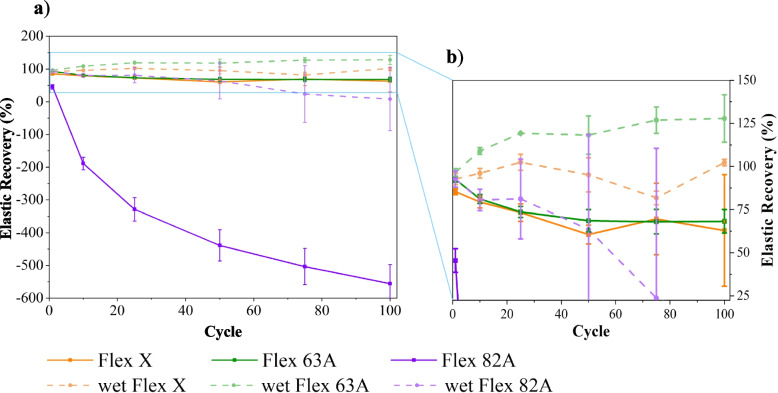
Elastic recovery (ER) of dry and wet specimens of Flex X, Flex 63 A, and Flex 82 A during cyclic tensile tests. **a** Results for all the specimens, and **b** detailed information for Flex X and Flex 63A

The dynamic performance of materials was assessed towards cyclic tensile tests (Fig. 4). Triplicates of each resin were tested, and the influence of storage condition (immersion in PBS – wet specimens) was also evaluated (Fig. 4 d)-f)). None of the specimens showed failure during the cyclic test or afterwards, which establishes their fatigue resistance and suitability for dynamic applications, such as mimicking biological tissues [69]. The elastic recovery ($ER$) along the cyclic test was calculated through $ER\left(\%\right)=\left(1-\frac{{\varepsilon }_{0}-{\varepsilon }_{i}}{{\varepsilon }_{0}}\right)\times 100\%$, where ${\varepsilon }_{0}$ and ${\varepsilon }_{i}$ are the tensile strains at the beginning of 1 st cycle and at the end of i^th^ cycle, respectively. $ER$ was determined at the 1 st, 10th, 25th, 50th, 75th and 100th cycles, and the results were plotted against the cycles (Fig. 5).

Stress–strain curves (Fig. 4) emphasize the conclusions inferred about the mechanical properties of the resins. For the same interval of imposed stresses, Flex X specimens exhibit higher tensile strain values due to their elasticity, while Flex 82 A has associated inferior tensile strain values, highlighting its superior elastic modulus. It is also observed a small stiffening after the first load cycle (confirmed by the enhancement of stress–strain slope) (Fig. 4 a)-c)), which may be attributed to micromechanical phenomena, e.g., “wear that can cause flaw blunting and ameliorate the stress considerations” [70], or due to the creation of residual deformation inside the specimens [71].

The reduction of Flex 82 A modulus is also noticeable upon the immersion of specimens in PBS solution (Fig. 4 c), f)). This was already confirmed in the static tensile test and attributed to the plasticising effect of water, which is often related to an increase in the free volume of the polymer, thereby increasing the mobility of the polymeric chains and, comparatively with the dry state, enhancing $ER$ (Fig. 5) [70]. The immersion of Flex X and Flex 63 A specimens in PBS solution also increased the $ER$ (Fig. 5). By sharing the same basic chemical composition and the lowest values of hardness, these two polymers undergo a higher percentage of elastic deformation with each tensile cycle. Consequently, the permanent deformation associated with the viscoelastic behaviour is smaller, and the sum after the 100th cycle is also smaller and very similar to the values obtained in the dry state. Moreover, the establishment of a hydrogen bond network can also significantly contribute to cohesion and enhance the polymer’s elastic recovery [72–74].

Hysteresis is another parameter to be analysed [75]. It was significant for dry Flex 82 A specimens, which may result from the plastic deformation of the hard segments of this polymer [69, 76, 77]. Flex X and Flex 63 A specimens (Fig. 4 a), b), d) e)), and wet Flex 82 A (Fig. 4 f)) specimens, however, present cyclic stress–strain loops showing little hysteresis, with a linear profile and a leaf-like appearance with a pointed tip. This behaviour is associated with small plastic deformation and a rapid response of elastic deformation [71]. Nonetheless, all the specimens (Fig. 4, a)-f)) display shifted hysteresis loops, which indicates the presence of creep. The creep behaviour is a sign of viscoelasticity [71], and it is translated by the loss of elastic recovery (Fig. 5).

**Fig. 6:**
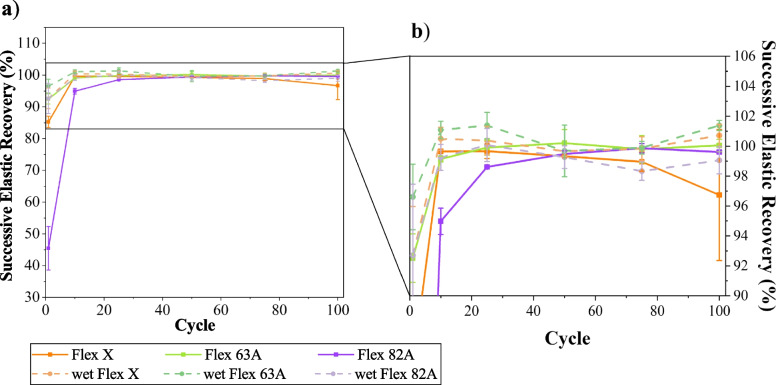
Successive recovery (SR) of dry and wet specimens of Flex X, Flex 63 A, and Flex 82 A, during cyclic tensile tests. **a**) Results for all the specimens, and **b**) detailed information for Flex X and Flex 63 A, and wet Flex 82A

To understand the creep behaviour, the recoverability through successive cycles ($SR$) was assessed (Eq. 5).

5 $$S{R}_{i} \left(\%\right)=\left(1-\frac{{\varepsilon }_{i-1}-{\varepsilon }_{i}}{{\varepsilon }_{i-1}}\right)\times 100\%$$

where ${\varepsilon }_{i-1}$ and ${\varepsilon }_{i}$ represent the tensile strain at the end of ${\left(i-1\right)}^{th}$ and ${i}^{th}$ cycles, respectively. The $SR$ values were determined for the 1 st, 10th, 25th, 50th, 75th and 100th cycles, and the results were plotted (Fig. 6). At the first cycle $SR$ is small, becoming more stable in the following cycles. This evolution could be ascribed to the elimination of the internal stresses of the materials in the first tensile cycle [78]. This implies that to be practically applied, these materials must be relieved of their residual stresses.

Nevertheless, the envisaged purpose of materials is their application on vascular biomodels subjected to pulsatile blood flow. Hence, their elastic recovery must be close to 100%. This value is nearly obtained for both dry and wet specimens of Flex 63 A and Flex X (Fig. 5, Fig. 6), confirming their suitability for the intended application. The recovery is more stable for the specimens immersed in PBS for 15 days, which corroborates the cohesive role of water [73]. Even so, caution must be taken when evaluating the results because the unloading strain rate could not have been sufficiently low to allow the stress-relaxation of the polymers and to permit the recoverability of their initial geometries. Indeed, some publications have noted that creep strain can be fully removed after load removal [70, 79].

**Fig. 7:**
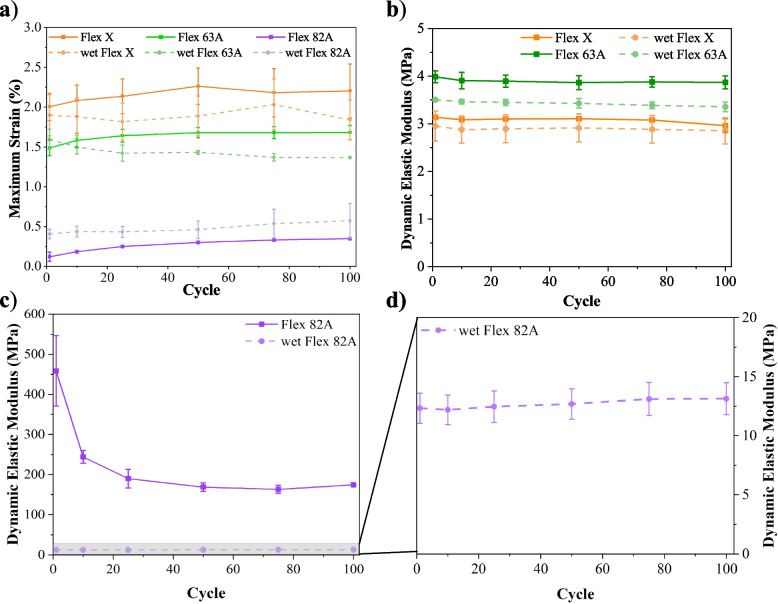
Results of dynamic tensile tests for dry and wet specimens of Flex X, Flex 63 A and Flex 82A. **a**) Maximum strain along the cyclic tests. **b**)-**d**) Relationship between the dynamic elastic modulus and the cycle number: **b**) Flex X and Flex 63 A, **c**) Flex 82 A, and **d**) detailed information for wet Flex 82A

The dynamic tensile performance was also assessed by determining dynamic elastic modulus (${E}_{d}$) for the 1 st, 10th, 25th, 50th 75th and 100th cycles (Fig. 7 b)-d)). This parameter was calculated using the approach of Chen and coworkers [71], as outlined in Eq. 6.

6 $${E}_{d}=\frac{{\sigma }_{max{i}_{i}}-{\sigma }_{min{i}_{i}}}{{\varepsilon }_{max{i}_{i}}-{\varepsilon }_{min{i}_{i}}}$$

where ${\sigma }_{max{i}_{i}}$ and ${\sigma }_{min{i}_{i}}$ are the maximum and minimum of stress in the i^th^ cycle, and ${\varepsilon }_{min{i}_{i}}$ and ${\varepsilon }_{min{i}_{i}}$ are the maximum and minimum of strain in the i^th^ cycle, respectively.

Apart from dry Flex 82 A specimens, all materials exhibit a stable elastic modulus, which confirms their dynamic resistance, and thus their appropriateness for cyclic stress applications. ${E}_{d}$ values are similar to the respective determined Young’s moduli (Table 3), meaning that cyclic tensile stress does not affect the mechanical properties of materials. However, for dry Flex 82 A specimens, this similarity did not occur, which indicates their sensitivity for dynamic applications (Fig. 4 c), Fig. 5 a), Fig. 6, Fig. 7 c)). The reduction in dynamic elastic modulus and consequent reduction in elastic recovery confirm the generation of irreversible deformation as the cycle number increases. For dry Flex 82 A, the decrease of ${E}_{d}$ (Fig. 7 a)) does not follow a linear profile, which means that the rate of plastic deformation decreases with the increase of cycle number [71].

### Shore a hardness

**Fig. 8:**
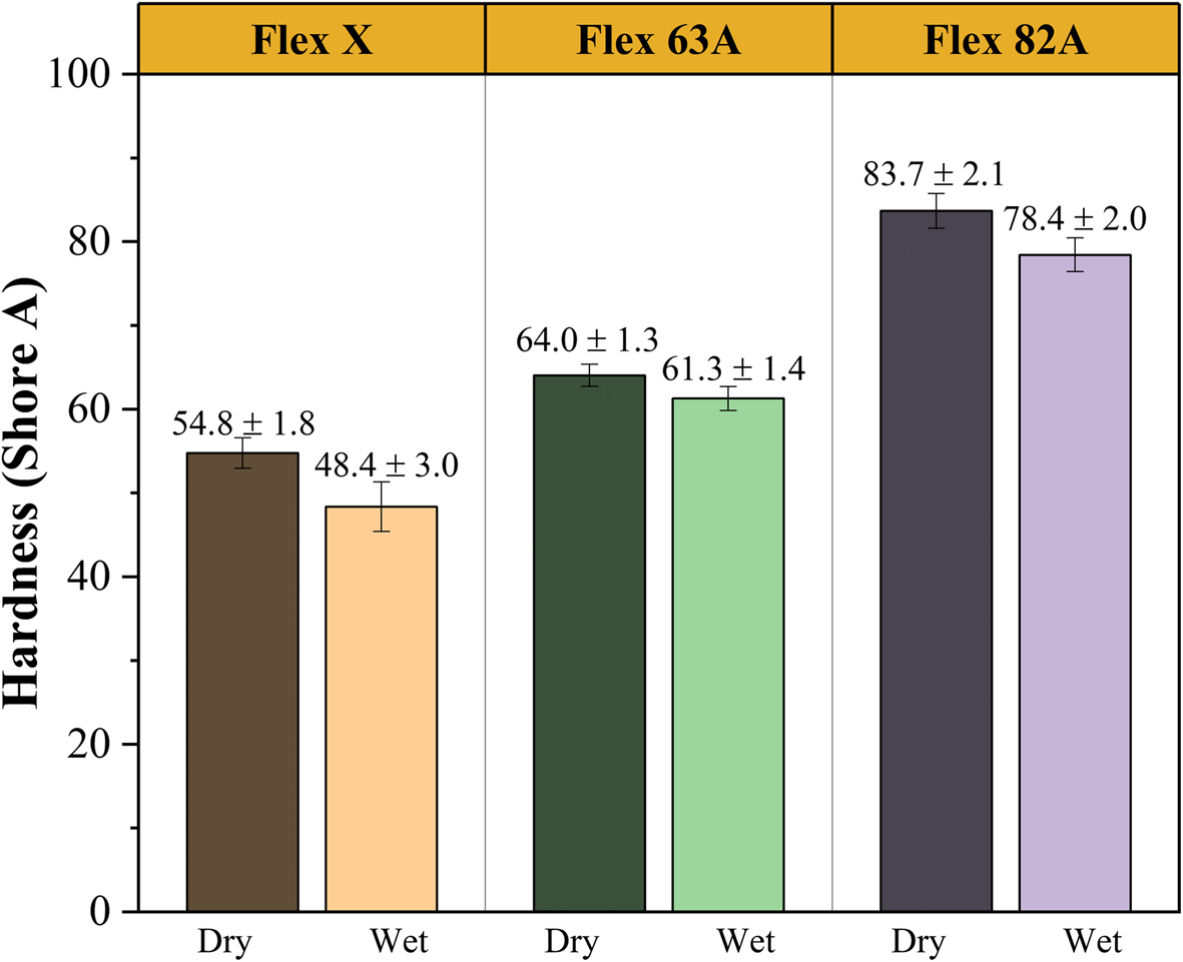
Shore A hardness values of the post-printed materials, comparing two different storage conditions (dry and wet). Results are presented as mean ± SD

The hardness values of the post-printed resins were measured using Shore A methodology [80], and the results are presented in Fig. 8. The experiments were conducted on both dry and wet specimens to evaluate the impact of the wetting process on the hardness property. The wet specimens were immersed in PBS solution for 15 days to ensure they reached maximum swelling capacity.

The results of dry specimens are in agreement with the expected Shore A hardness values provided by the suppliers: 54.8 ± 1.8 Shore A vs 55 Shore A [43], 64.0 ± 1.3 Shore A vs 63 Shore A [44], and 83.7 ± 2.1 Shore A vs 82 Shore A [45], for Flex X, Flex 63 A, and Flex 82 A, respectively. The differences among the polymers are due to their chemical composition. The literature describes the effect of acrylate content on the hardness of materials [62], citing that higher acrylate contents result in harder materials. By analysing the chemical composition of resins, it can be confirmed that Flex X [43] and Flex 63 A [44] have lower amounts of acrylate-derived components, whereas Flex 82 A has a higher percentage of this precursor (25 to 50% (w/w) of 2-hydroxypropyl – HPMA) [45]. Moreover, longer oligomers, such as urethane acrylate oligomers, produce longer polymeric chains and, thus, softer materials [62, 65], which explains the lower Shore A hardness values for Flex X and Flex 63 A resins.

It is also worth mentioning a reduction of the Shore A hardness values for the specimens immersed in PBS solution. Hardness is the mechanical resistance of a material to plastic deformation by an indenter, measuring the stiffness obtained from permanent penetration [81, 82]. Therefore, higher values suggest stiffer materials. Once again, the presence of aqueous solution in the bulk increases the plasticity of the material, decreasing the elastic part of its response [61]. Hence, its resistance to indentation declines and lower hardness values are instigated.

To tailor the hardness values, specimens were irradiated with ultraviolet light (λ = 254 nm, UV-C henceforth) to observe any differences in the results. Theoretically, UV radiation may alter the mechanical properties by degrading the polymeric chains [83, 84]. Two irradiation times were compared (30 and 60 min), and each condition was tested duplicate five times. The results are presented in Table 4.

**Table 4:** Shore A hardness of the thermosetting polymers after being exposed to UV-C for 30 and 60 min, and comparison with the control condition

Material	Exposure Time of UV-C	Shore A Hardness
Flex X ^1,2^	Control	54.8 ± 1.8
	30 min	51.5 ± 1.5
	60 min	52.7 ± 1.8
Flex 63 A ^1,2,3^	Control	64.0 ± 1.3
	30 min	53.3 ± 1.7
	60 min	58.4 ± 3.3
Flex 82A	Control	83.7 ± 2.1
	30 min	83.8 ± 2.3
	60 min	85.8 ± 2.8

Statistically significant differences between: (1) the control group and the group of 30 min of exposure; (2) the control group and the group of 60 min of exposure; and (3) the group of 30 min of exposure and the group of 60 min of exposure. Results were derived from the one-way ANOVA test, using the Holm-Sidak method to compare groups (p-value < 0.05)

From the results, it can be concluded that, for Flex 82 A specimens, UV-C irradiation does not introduce any statistically significant difference, which is likely due to the chemical nature of the polymeric network, which is more stable under UV-C irradiation. All three initial resins contain methacrylate-derived oligomers; however, Flex X and Flex 63 A are composed of carbamate groups, which have additional ester bonds that are photochemically unstable [85], whereas Flex 82 A does not. This polymer has more ether bounds, and it has been established that these chemical bonds increase both the toughness and UV stability of the polymers [86], which corroborates the obtained results.

For Flex X and Flex 63 A, results indicate that the UV-C exposure reduces the hardness of the specimens. Moreover, for longer exposure time, a slight increase in Shore A hardness is observed, which suggests a two-step interaction between UV radiation and the polymeric chains. The first 30 min were associated with the breakage of covalent bonds within the chains and the consequent reduction in molecular weight, which translates into the first decline in hardness values [87, 88]. The literature reports that cross-linked polyester resins are photochemically unstable, and upon irradiation with UV light, carbonyl groups are formed, potentiating the decrease of polymeric molecular weight [85]. Since Flex X and Flex 63 A are polyester-based thermosetting polymers (Fig. 2 b)-c)). The decline in hardness values after 30 min of UV-C exposure is explained.

Meanwhile, the partial disintegration of ester-based chains and the oxidation of phenolic hydroxyl groups lead to the formation of free radicals and carbon monoxide (CO) and carbon dioxide (CO_2_) molecules, respectively [89]. Free radicals undergo recombination with neighbouring groups, triggering the branching and cross-linking of the polymeric structures. This rearrangement of the polymeric chains instigates mechanical strength, resulting in higher hardness values [85] after 60 min of UV-C exposure.

Hence, in the first 30 min of irradiation, the balance between photodegradation and photooxidation favours degradation, producing softer specimens. In the final phase of UV-C irradiation, the reorganisation of polymeric chains and their branching are facilitated, resulting in higher hardness values. Therefore, UV irradiation could be an interesting candidate for fine-tuning the hardness of polymeric materials and, thus, obtaining a suitable cardiovascular mimicking material.

As reported, Shore A hardness can be used to measure in vivo biomechanics [90], and it has been used to characterize vascular tissues [91, 92]. Comparisons with literature have shown that the studied materials have Shore A hardness values within the range used to mimic vascular tissues (26–28 Shore A) and calcifications (68–72 Shore A) [30], indicating their suitability for manufacturing atherosclerotic physical models.

Some works [64, 93] report the selection of biomimicking materials using a mathematical expression (Eq. 7) that relates the Shore A hardness of the material with the elastic modulus of the biological tissue, which in turn must be similar to the elastic modulus of biomimicking material.

7 $$\text{log}{E}_{0}=0.0235 \times S-0.6403$$

where ${E}_{0}$ is the estimated elastic modulus, and $S$ is Shore A if 20 A < S <  80 A, or Shore D + 50 if 80 A < S < 85D. In order to investigate the pertinence of this empirical relation, ${E}_{0}$ values were determined from the Shore A hardness values previously measured (Table 4) and compared with the respective $E$ (Table 3). Similarly, a mathematical formulation was obtained that fits the experimental results of the present work. With this aim, the logarithm of $E$ values (Table 3) were plotted against the respective Shore A hardness, and a linear relationship ($r=\text{0,9012}$) was found (Eq. 8).

8 $$\text{log}E=0.0321 \times S-1.343$$

where $E$ is the Young’s modulus obtained in static tensile test and $S$ the respective Shore A hardness. Projected elastic moduli (${E}_{p}$) were calculated using Eq. 8 and compared with ${E}_{0}$ and $E$ to ensure the validity of this formulation, and results are shown in Table 5.

**Table 5:** Estimated elastic moduli of 3D printed specimens (${E}_{0}$), and projected elastic moduli (${E}_{m}$) using Eq. 7 and Eq. 8, respectively. The calculation was done for post-processed specimens (control), wet specimens (previously immersed in PBS solution for 15 days), and irradiated specimens with UV-C light for 30 min (UV-C_30 min) and 60 min (UV-C_60 min)

Material	Testing Condition	Shore A Hardness	Young’s Modulus${\varvec{E}}$(MPa)*	Estimated${{\varvec{E}}}_{0}$(MPa)**	Projected${{\varvec{E}}}_{{\varvec{p}}}$(MPa)***
Flex X	Control (Dry)	54.8	2.7	4.4	2.6
	Wet	48.4	2.2	3.1	1.6
	UV-C_30 min	51.5	___	3.7	2.0
	UV-C_60 min	52.7	___	4.0	2.2
Flex 63A	Control (Dry)	64.0	3.4	7.3	5.1
	Wet	61.3	3.9	6.3	4.2
	UV-C_30 min	53.3	___	4.1	2.3
	UV-C_60 min	58.4	___	5.4	3.4
Flex 82A	Control (Dry)	83.7	34.6	21.2	22.1
	Wet	78.4	11.5	15.9	14.9
	UV-C_30 min	83.8	___	21.3	22.2
	UV-C_60 min	85.8	___	23.8	25.7

^*^ Young’s moduli determined from stress–strain curves of tensile tests

** Estimated elastic modulus (${E}_{0}$), calculated using Eq. 7

*** Projected elastic modulus (${E}_{p}$), obtained from the linear regression model (Eq. 8) fitted to our results

Both the estimated and projected elastic moduli are by the respective Young’s moduli, which validates the linear relation between the logarithm of elastic modulus and hardness scale [64]. ${E}_{p}$ values are closer to the determined Young’s moduli ($E$), as expected. However, the overestimation of ${E}_{0}$ and ${E}_{p}$ compared with $E$, confirm the empirical nature of the mathematical expressions.

The estimation of Young’s modulus of biological tissues using other characterisation techniques has been extensively explored [94–98]. For example, the force-indentation equation was applied to nanoindented atherosclerotic tissues, and the estimated Young’s modulus ranged from 3 to 105 kPa, which are 1000 times lower than $E$, ${E}_{0}$ and ${E}_{p}$ of the studied materials [94, 95]. On the other hand, other works derived the elastic modulus of atherosclerotic plaques from other indentation models, and results are widely dispersed: 21–25 GPa [96], 17–28 GPa [97], 20–23 GPa [98], six orders of magnitude higher than Rezvani-Sharif results [94, 95]. Together, these studies corroborate the variability of the biomechanical results; however, as the characterisation methods differ, direct comparisons should not be made.

### Surface characterization

**Fig. 9:**
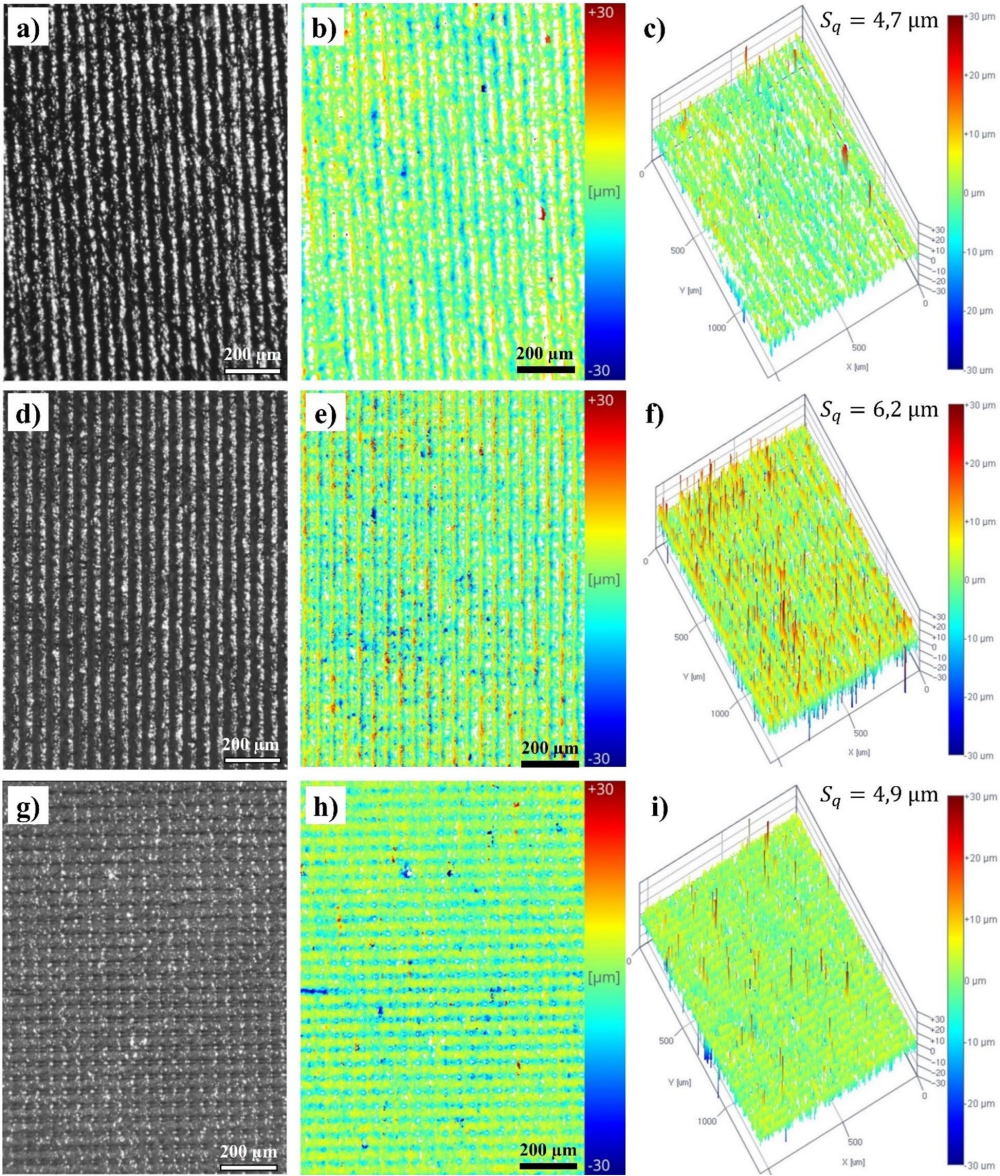
Topographic images of Flex X (**a**)—**c**)), Flex 63 A (**d**)—**f**)), and Flex 82 A (**g**)—**i**)) specimens from the OneAttension® Tensiometer, using the fringe projection phase-shifting method. Optical images (**a**), **d**), **g**) with 200 µm scale), 2D roughness maps (**b**), **e**), **h**) with 200 µm scale), and 3D roughness maps (**c**), **f**), **i**)), were acquired for each specimen

The contact angles (${\theta }_{a}$) of Flex X, Flex 63 A and Flex 82 A, before the correction with Wenzel’s equation (Eq. 4), were determined to be 94.7 ± 2.6°, 133.1 ± 5.4° and 91.6 ± 6.2°, respectively. It is well known that roughness strongly influences the measurement of static contact angle [99, 100], so the corrected contact angles were determined accordingly. This correction utilised the *r* factor and was achieved by analysing the surface roughness at the same position where the contact angle is measured [101]. The corrected contact angles (${\theta }_{r}$) were calculated to be 92.4 ± 1.6°, 104.0 ± 1.4° and 90.2 ± 1.1°, for Flex X, Flex 63 A and Flex 82 A, respectively. Representative 2D and 3D roughness maps of each UV-curable material are presented in Fig. 9. The tensiometer pre-acquired optical images, so the roughness parameters can be estimated using the fringe projection phase-shifting method. The optical images and 2D and 3D roughness maps reveal the inherent 3D printing paths, specifically the pixels of the LCD used to photo-crosslink the resins during UV exposure.

The contact angle calculation allows the determination of hydrophilicity.

(${\theta }_{r}\le 65^\circ )$ or hydrophobicity (${\theta }_{r}\ge 65^\circ )$ of the printed specimens. As all the specimens have contact angles above 65°, it can be stated that they are hydrophobic [102]. Due to the presence of the -OH group on the surface of Flex X and Flex 82 A, their contact angles are smaller than that of Flex 63 A, which does not show this polar group on its FTIR spectrum [103].

Although some literature asserts that hydrophilic surfaces are favourable for guaranteeing protein absorption and promoting vascular regeneration, the intended application does not require this parameter [104]. Indeed, a more important requirement is the inhibition of bacterial attachment. The effect of contact angle on bacterial proliferation was studied [105], and it has been proved that hydrophobic surfaces, with contact angles of about 100°, inhibit bacterial adhesion. The work [105] further concluded that even low flow rates could hamper bacterial adhesion, but surfaces must be hydrophobic.

Moreover, it has been established that hydrophobic surfaces allow sequential flows for bioassay applications and capillary-driven microfluidic devices [106]. Microfluidics refers to systems that utilise microtubes to manipulate fluids [107], which is similar to the capillary behaviour of small arteries, such as coronary arteries.

Regarding the effect of roughness on the results, it was concluded that the studied surfaces presented a $r$ factor values of 1.54, 2.81, and 2.09 for Flex X, Flex 63 A, and Flex 82 A, respectively. As stated in the literature, for the hydrophobic regions, as the roughness increases, the apparent contact angle increases, and the flow resistance decreases [100]. This phenomenon can be attributed to the accumulation of gas bubbles on the valleys of rough surfaces, which transfers the boundary condition from a non-slip to a slip condition, thereby promoting fluid flow [108]. The resistance to fluid flow is especially important in vascular models, as it enable them to mimic real blood flow conditions and replicate the functionality of flow mechanics [109]. For this reason, the studied surfaces are suitable for producing vascular structures in which blood-like fluid flows inside them.

### Morphological characterization

**Fig. 10:**
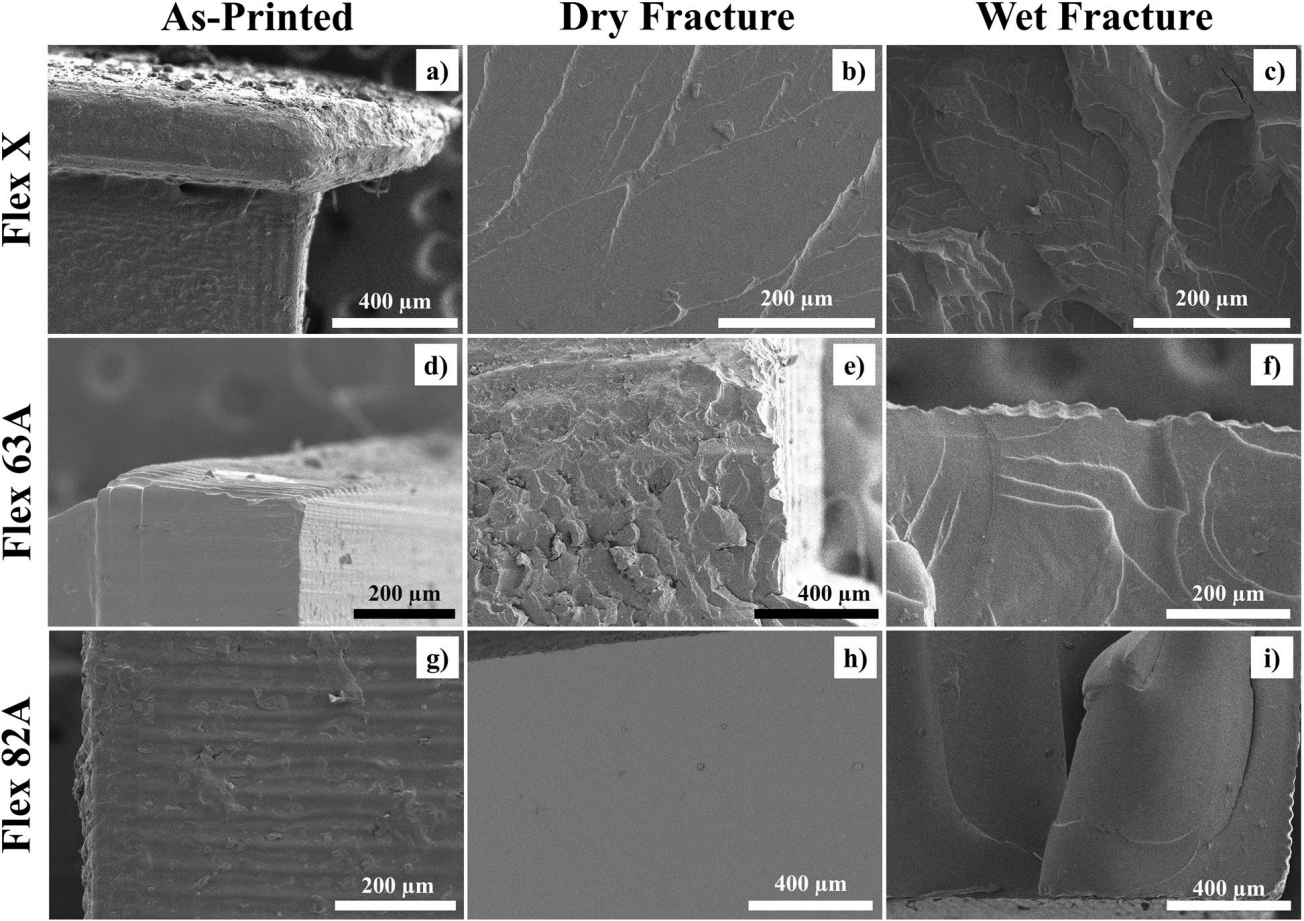
SEM micrographs of **a**), **b**) and **c**) Flex X; **d**), **e**) and **f**) Flex 63 A; **g**), **h**) and **i**) Flex 82 A resin specimens. Analysed specimens correspond to **a**), **d**) and **g**) post-printed specimens; **b**), **e**) and **h**) dry fracture; **c**), **f**), and **i**) wet fracture. Scale bars **a**), **e**), **h**), and **i**) = 400 µm; **b**), **c**), **d**), **f**), and **g**) = 200 µm

The surface morphology of post-printed specimens of Flex X, Flex 63 A, and Flex 82 A was observed using SEM, and the micrographs are presented in Fig. 10 a), d), g). Likewise, the ruptured surface of specimens that were subjected to tensile tests were also analysed, and their respective micrographs were obtained (Fig. 10 – b), e), h)). The storage condition (immersion into PBS solution) was evaluated, and the fracture of specimens after the tensile test was also assessed (Fig. 10 – c), f), i)).

The quality of 3D printed parts seemed satisfactory upon visual inspection for all the studied resins. However, scanning electron micrographs revealed a staircase effect on all three materials, as depicted in Fig. 10 – a), d), and g). Although SLA has better printing resolutions than other additive manufacturing processes and technologies, it also produces parts in which the slicing of each printed layer is visible, confirming that this is a layer-by-layer deposition technology [110]. Likewise, a larger border was observed for all the resins, exemplified by the specimen Flex X (Fig. 10 – a)), which corresponds to the first deposited layers, where a longer exposure time was applied to ensure adhesion between the polymerized resin and the printing bed.

Regarding the ruptured surfaces, it can be stated that for all the conditions, fractures were fragile. Damage patterns were similar, and microscale cracks were formed with the production of in-plane failure [65]. However, the effect of water was noticeable in the fracture morphology of wet specimens, which presented blunter edges (Fig. 10 – f)). The fracture along the delamination planes, resulting from the layer-by-layer construction, was caused by plasticization by water (Fig. 10 – i)).

## Conclusions

Three resins were processed by SLA, and the resulting thermosetting polymers were chemically, physically, topographically and mechanically characterized to investigate the feasibility of their use in cardiovascular physical modelling. The reported results support the use of any UV-curable resin to produce atherosclerotic vascular models, although it is challenging to interpret and compare them with the results of biological tissue properties. The numerous characterisation methodologies used to assess the biomechanical properties of tissues and the variability introduced by donor-specific anatomical and pathological conditions are the source of widely dispersed values [23, 25, 59].

Nonetheless, it was noted that 3D printing offers the possibility to reproduce the anatomical and structural variations of the vascular system by implementing materials that can mimic vessel mechanical properties. Indeed, as the studied materials exhibit distinct mechanical behaviours, their combination could be an interesting approach to adjust and tune the final properties, thereby achieving a more reliable human model with properties ranging from soft and compliant to stiff and atherosclerotic. This may be accomplished using Flex X as the softer material and Flex 63 A and Flex 82 A as the atherosclerotic-mimicking materials. Additionally, the PBS solution was used to simulate the human vascular environment and the immersion of materials in it, causing their “softening” and resulting in more similar physiological performance.

Moreover, since vascular structures are compliant and undergo elastic deformation in response to pressure variations [111], ideal 3D printed biomodels must possess this characteristic and exhibit fatigue resistance for long-term use. For the polymers studied in this work, the parameter was validated through cyclic tensile tests. Results demonstrated that all the wet specimens were fatigue resistant and presented a high elastic recovery, which is advantageous to manufacturing vascular structures.

The tunability of the mechanical properties of specimens was demonstrated to be achieved through the use of UV-C radiation. However, complementary analysis should be conducted. Another tuning approach could be the chemical modification of the initial resins by adding softener or hardener compounds. This strategy has been tested [112] to obtain customised materials capable of mimicking biological tissues; however, the work did not utilise additive manufacturing.

In conclusion, this work confirms that commercial UV-curable resins could be used to mimic atherosclerotic materials. The mechanical properties can be adjusted by irradiating post-printed structures with UV-C light or by simply immersing them in PBS solutions. Waste residues and production costs are challenges that were overcome because additive manufacturing was directly implemented. Additionally, commercial resins were used, eliminating the need for polymeric synthesis, which simplifies the manufacturing process. Therefore, this work proved to be a remarkable preliminary study addressing the biomimicking of atherosclerotic blood vessels.

## Data Availability

No datasets were generated or analysed during the current study.
